# Genome-wide association analysis suggests novel loci underlying thyroid antibodies in Hashimoto’s thyroiditis

**DOI:** 10.1038/s41598-019-41850-6

**Published:** 2019-03-29

**Authors:** Luka Brčić, Ana Barić, Sanda Gračan, Vesela Torlak, Marko Brekalo, Veselin Škrabić, Tatijana Zemunik, Maja Barbalić, Ante Punda, Vesna Boraska Perica

**Affiliations:** 1 Department of Medical Biology, University of Split, School of Medicine, Split, Croatia; 2 Department of Nuclear Medicine, University Hospital Split, Split, Croatia; 3 Department of Pediatrics, University Hospital Split, Split, Croatia

## Abstract

Thyroid antibodies against thyroglobulin (TgAb) and thyroid peroxidase (TPOAb) are key markers of Hashimoto’s thyroiditis (HT), the most common autoimmune thyroid disorder. Genetic determinants of thyroid antibodies are still poorly known, especially as they were not studied in patients with thyroid diseases. We performed the first genome-wide association analysis of thyroid antibodies in 430 HT patients that may be considered as population extremes for thyroid antibodies distribution. We detected two suggestively associated genetic variants with TgAb, rs6972286 close to *ANKRD7* and *LSM8* (P = 2.34 × 10^−7^) and rs756763 inside *CA10* (P = 6.05 × 10^−7^), and one with TPOAb, rs12507813 positioned between *TRIM61* and *TRIM60* (P = 4.95 × 10^−7^). Bivariate analysis resulted with three suggestively associated genetic variants that predispose to both antibodies: rs13190616 inside *RP11-138J23.1* (P = 2.01 × 10^−6^), rs561030786 close to *DUBR* (P = 7.33 × 10^−6^) and rs12713034 inside *FSHR* (P = 7.66 × 10^−6^). All identified genomic regions have a substantial literature record of involvement with female-related traits, immune-mediated diseases and personality traits that are all characterized by increased thyroid antibody levels. Our findings demonstrate the existence of genetic overlap between thyroid autoimmunity in HT and different non-thyroid diseases characterized by the presence of thyroid antibodies. We also suggest that genetic variants that regulate antibody levels may differ between HT patients and individuals with normal thyroid function.

## Introduction

Hashimoto’s thyroiditis (HT), also known as chronic lymphocytic thyroiditis or autoimmune thyroiditis, is the most common autoimmune thyroid disorder (AITD) characterized by the infiltration of lymphocytes in the interstitium among the thyrocytes and destruction of thyroid tissue^1,2^.

Another distinguished feature of HT is the presence of elevated thyroid antibodies against two major thyroid antigens - thyroglobulin (Tg) and thyroid peroxidase (TPO)^3^. Tg is a large homodimeric glycoprotein synthesized by thyrocytes and secreted into the follicular lumen^4^. It is involved both in the synthesis and in the storage of thyroid hormones - triiodothyronine (T3) and thyroxine (T4)^4^. TPO is a homodimeric membrane-bound enzyme also involved in the synthesis of thyroid hormones^5^. TPO plays a role in the formation of monoiodotyrosine (MIT) and diiodotyrosine (DIT) as it catalyzes iodination of thyrosine residues. TPO also catalyzes coupling of MIT and DIT in Tg, which results in the formation of T3 and T4^6^.

Although Tg and TPO are different antigens, antibodies against Tg (TgAbs) and TPO (TPOAbs) have many common features^7^. Both TPOAb and TgAb are mostly of immunoglobulin (Ig) G class and exhibit high affinities for their antigens^7^. TPOAbs are present in about 90% and TgAbs in about 80% of HT patients^7,8^. Both thyroid antibodies serve as key clinical markers for detection of HT, however TPOAbs are considered as best predictors for thyroid autoimmunity^1^. Although thyroid antibodies are markers for thyroid damage, it is considered that they are not causative factors and most likely do not have a key role in the pathogenesis of HT^8,9^. Nevertheless, it is known that TPOAbs can fix complement and therefore cause further damage to thyrocytes by an antibody-dependent cell cytotoxic mechanism^7,10^. However, it is most likely that the TPOAb-dependent cell cytotoxicity does not have such high impact in overall thyroid destruction as T-cell mediated cytotoxicity does^8^. On the other hand, it is considered that TgAbs do not cause any damage to thyroid tissue^5^.

Thyroid autoimmunity is not solely associated with thyroid diseases but also with other common autoimmune and non-autoimmune diseases^5^. In general, circulating TgAb and TPOAb antibodies are detected in 9–25% of non-AITD patients^11^, but are higher in frequency among patients with autoimmune diseases such as rheumatoid arthritis^12^, type 1 diabetes^13,14^, celiac disease^15^, polycystic ovary syndrome (PCOS)^16,17^, systemic lupus erythematosus (SLE)^18^, and other diseases such as asthma^19–21^ and urticaria^18^. A role of thyroid antibodies is also suggested with mood disorders, anxiety and depression^22^. Finally, of the malignancies, one of the most investigated associations is between thyroid antibodies and breast cancer (BC) where several studies pointed out that thyroid autoimmunity confers a greater risk for development of BC^23–26^.

Thyroid antibodies have strong genetic background and it has been estimated that genetic factors contribute to about 70% of the susceptibility to develop thyroid antibodies^27^. So far, there were three genome-wide association studies (GWAS) that investigated genetic variants associated with TPOAb levels/positivity in general population; two of these studies were performed in European population^28,29^ and one was performed in Korean population^30^. Altogether, these studies identified several TPOAb-associated loci such as *TPO, ATXN2, MAGI3, BACH2, HLA-DOB, HLA-DPB1, HCP5, RERE* and *KALRN*^28–30^. It was also shown that individuals with a high genetic risk score, based on these loci, had higher TSH levels, lower T4 levels and increased risk of hypothyroidism^29^. Recently, GWAS of both thyroid antibodies, performed in general Croatian population, discovered novel female-specific association near *GRIN3A* with both TgAb and TPOAb levels^31^. Additionally, one GWAS of TPOAb levels was performed in patients with type-1 diabetes (T1D)^32^. This study suggested several T1D susceptibility genes that also contribute to TPOAb levels, including *STAT4, BACH2, UBASH3A, PTPN22, CTLA4, IL2, RASGRP1, SH2B3, FCRL3, FCRL1, IL2-IL21* and *SPPL3*^32^, pointing that there are possibly more undiscovered TPOAb and TgAb loci that are also involved in the development of other non-thyroid diseases characterized by the presence of thyroid antibodies.

Although many associations have been identified, genetic determinants of thyroid antibodies are still not fully understood, especially as they were not studied in patients with thyroid diseases, such as HT. For that reason we performed GWAS analysis of TPOAb and TgAb levels in a cohort of 430 HT patients with the aim of identifying genetic variants associated with thyroid antibodies in this pathologic condition. Thyroid antibodies are markers of HT and are generally increased in HT patients thus making our sample more powerful for detection of underlying genetic variants. The main focus of our study is to identify TPOAb/TgAb-associated genetic variants that are enriched in HT, as opposed to variants that have already been established for general population or T1D patients. This is the first GWAS that investigated genetic background of thyroid antibodies levels on genome-wide scale in patients with HT.

## Methods

### Subjects

A total of 430 HT patients including 400 females (93%) and 30 males (7%) were involved in this study. Patients were recruited at the Department for Nuclear Medicine at the University Hospital Split (Croatia) from 2013 to 2016. All patients were of white European ancestry, adult and living in the south Croatia, a region considered iodine sufficient since 2003^33^. Diagnosis of HT followed ETA recommendations and guidelines for Management of Subclinical Hypothyroidism^34^. Diagnosis was established primarily on the basis of clinical examination, thyroid hormone values, positivity to thyroid antibodies and characteristic thyroid ultrasound (US) image (unhomogenic and/or hypoechogenic thyroid tissue). Demographic and clinical attributes of participants are shown in Table 1.

**Table 1:** Clinical characteristics of HT patients.

Variable	Median (Q1–Q3)	Referent values
Age, years	37,8 (28,2–48,3)	/
TgAb, IU/ml	135,5 (39,3–428)	5–100
TPOAb, IU/ml	205,5 (27,45–639)	1–16
TSH, mIU/L	3,23 (1,76–5,2)	0.3–3.6
T3, nmol/L	1,7 (1,4–1,8)	1.3–3.6
T4, nmol/L	106 (89,78–119)	57.4–161
fT4, pmol/L	12,1 (10,7–13,4)	10.1–22.3

Q1-first quartile, Q3-third quartile.

Thyroid hormones and antibodies levels in plasma of HT patients were determined by an immunoassay method. Immunoassay reaction was conducted in a fully automated instrument “Liaison” Biomedica Chemiluminescence Analyzer in the Laboratory of Biochemistry, University Hospital Split, using *in vitro* assays for the quantitative determination of thyroid hormones and antibodies levels. Thyroid ultrasonography was performed using Medison Accuvix V10 (Samsung Medison Co., Ltd, Seoul 135–280, Korea) high frequency linear probe (8–12 Hz).

Genomic DNA of HT patients was extracted from peripheral blood leukocytes, using Nucleon Genomic DNA Extraction Kit BACC3, according to the manufacturer’s instructions (GE Healthcare, Little Chalfont, Buckinghamshire, UK). DNA concentration was determined by the Nanodrop ND-1000 Spectrophotometer (ND-1000, Thermo Fisher Scientific, USA). The final concentration of the DNA was uniformed across all samples and brought to 100 µg/µl prior genotyping.

### Genotyping, quality control (QC) and imputation

All participants were genotyped using Illumina Infinium HumanCoreExome genotyping platform that contains 551 839 markers. We performed standard sample and single-nucleotide polymorphism (SNP) quality control (QC) to exclude low quality samples and SNPs that could potentially lead to bias in results.

In the sample QC we excluded all samples with call rate <95% and all samples with heterozygosity rate deviating more than three standard deviations from the mean. We compared reported sex of participants with the sex inferred from the genotypes - no exclusions were made based on this criterion. We also performed ethnicity check by visually inspecting multidimensional scaling analysis (MDS) plot and excluding outliers. We also checked for pair-wise identity by descent (IBD) to identify potential duplicate samples. There were no duplicate samples in our dataset, that is, each pair of samples had $\hat{\pi }$ < 0.9. After performing sample QC, we excluded a total of 25 samples, and selected remaining 405 HT patients for imputation and GWAS.

In the SNP QC, we excluded all variants with call rate <98% and all variants deviating from Hardy-Weinberg equilibrium (HWE) (P < 10^−4^).

Following QC, we performed genotype imputation. In order to produce best-guess haplotypes and to speed up the imputation process^35^, genotypes were firstly pre-phased using SHAPEIT2 software^36^ and then imputed using IMPUTE2 software^37^. We used 1000 Genomes as reference panel (Phase3). In the post imputation QC, we excluded variants with MAF ≤5%, variants with INFO metric less than 0.4 and variants with HWE P < 10^−4^. We also checked concordance tables to assess an overall imputation quality and evaluated the concordance rate and INFO (type 0) metric of directly typed SNPs to detect poorly genotyped SNPs and strand flips.

### Genome-wide association analyses

Association analyses between each of resulting 6 007 542 autosomal genetic variants and TPOAb/TgAb levels were performed using GEMMA software^38^. TPOAb and TgAb levels were firstly regressed on gender using R statistical software. Derived residuals were then quantile transformed in R to obtain normal distribution and used as new phenotypes in association analyses. Association analyses were performed under the linear mixed model implemented in GEMMA software which accounts for population stratification and relatedness^38^. Centered relatedness matrix was calculated using centered genotypes from a set of 244 333 directly genotyped autosomal markers utilizing GEMMA. Manhattan plots of the genome-wide association results were generated using R package qqman. We visually inspected cluster genotyping plots to eliminate spurious associations of our top associated SNPs (P < 10^−5^). We checked cluster plots of all directly genotyped SNPs with P-value less than 0.1 located +/−400 kb from associated SNP. Cluster genotyping plots were generated using Ilummina GenomeStudio software. For all top-associated SNPs we checked the distribution of TgAb and TPOAb levels per genotype and generated box plots using R statistical software.

### Power analysis

We performed the power estimation for single trait GWAS analyses using various scenarios. We estimated the power for detection of genetic variants with a range of effect sizes (from 0 to 1) and a range of minor allele frequencies (MAFs) (from 0.05 to 0.45) on the genome-wide level of significance. For each given effect size and MAF we ran 10 000 simulations of genotypes and phenotypes for 405 individuals. Genotypes (*X*), that is the number of minor alleles (0, 1 or 2), were generated from trinomial distribution dependent on MAF, with probabilities: $P(X=0)={(1-MAF)}^{2},\,P(X=1)=2(MAF)(1-MAF),\,P(X=2)=MA{F}^{2}$, to assure Hardy-Weinberg equilibrium. For given effect size (*β*), phenotypes were then generated from normal distribution dependent on genotype with mean *μ* = −*β* + *βX* and standard deviation $\sigma =\sqrt{1-{\beta }^{2}}$. For each simulation we performed association analysis using linear regression model and then estimated the power at a given site as a proportion of simulations with P value less than 5 × 10^−8^. Power analysis was performed using R statistical software.

### Bivariate GWAS analysis

As thyroid antibodies are strongly correlated in HT patients (r^2^ = 0.44 in our dataset), we also performed bivariate GWAS analysis, which tests the association between genetic variants and TPOAb and TgAb levels jointly, to increase the power of identifying loci associated with both thyroid antibodies levels. Bivariate analysis was performed using multivariate analysis of variance (MANOVA) method implemented in R package MultiABEL^39^. MANOVA test statistic was calculated using summary statistics from single GWAS of TPOAb levels and GWAS of TgAb levels. Cluster plots of top SNPs resulting from bivariate analysis were also checked, as described above.

### Analysis of results from previously published GWAS studies

We selected 7 SNPs previously associated with TgAbs in general population^31^ and 16 SNPs associated with TPOAbs in general population^28,30,31^ on the basis of P < 10^−6^ in original reports, and all 11 reported SNPs for TPOAbs in T1D patients^32^ and checked their associations in our GWAS results. We compared P-values and directions of effects from original reports with our summary statistics results and performed a sign test to assess the significance of the proportion of effects in the same direction. We also looked-up all SNPs located in +/−400 kb regions from originally-reported SNPs in our summary statistic results to check if there are additional associations.

### Compliance with Ethical Standards

Written informed consent was obtained from all study participants, and the study was approved by two separate Ethics Committees: the University of Split, School of Medicine (Classification no. 003-08/14-03/0001; Registry no. 2181-198-03-04-14-0028) and University Hospital Split (Classification no. 530-02/13-01/11; Registry no. 2181-147-01/06/J.B.-14-2). Both Ethics Committees declared that study is in accordance with the provisions of the Code of Ethics and the Helsinki Declaration.

## Results

### Genome-wide association analyses

**Figure 1:**
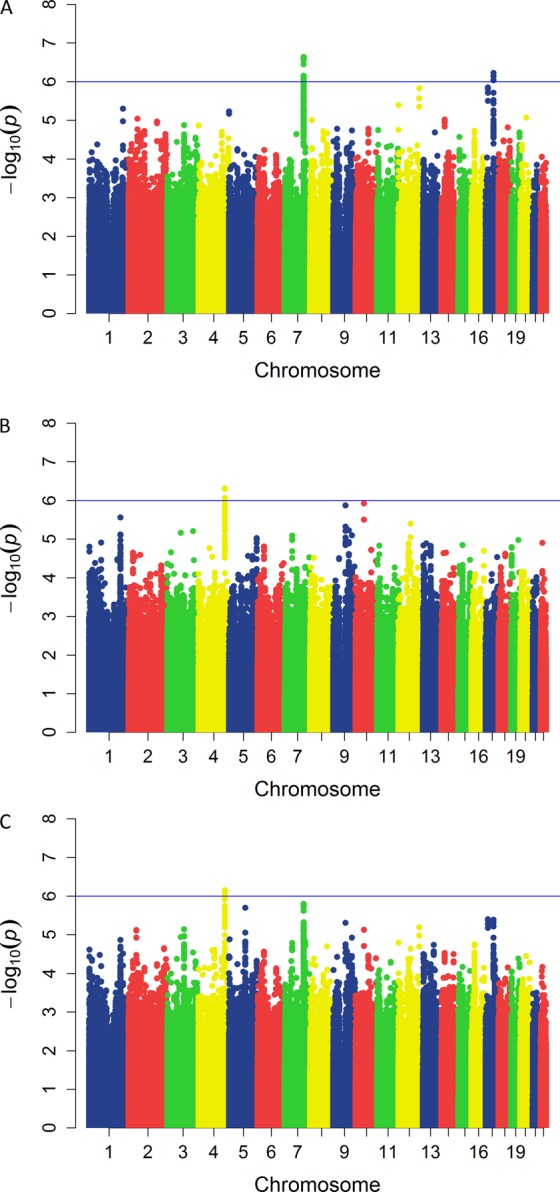
Manhattan plots of TgAb levels (**A**), TPOAb levels (**B**) and bivariate analysis (**C**). For each analyzed SNP, the x-axis shows chromosomal position, while y-axis shows the −log10(P) value. The blue line indicates the threshold of P = 10^−6^.

**Figure 2:**
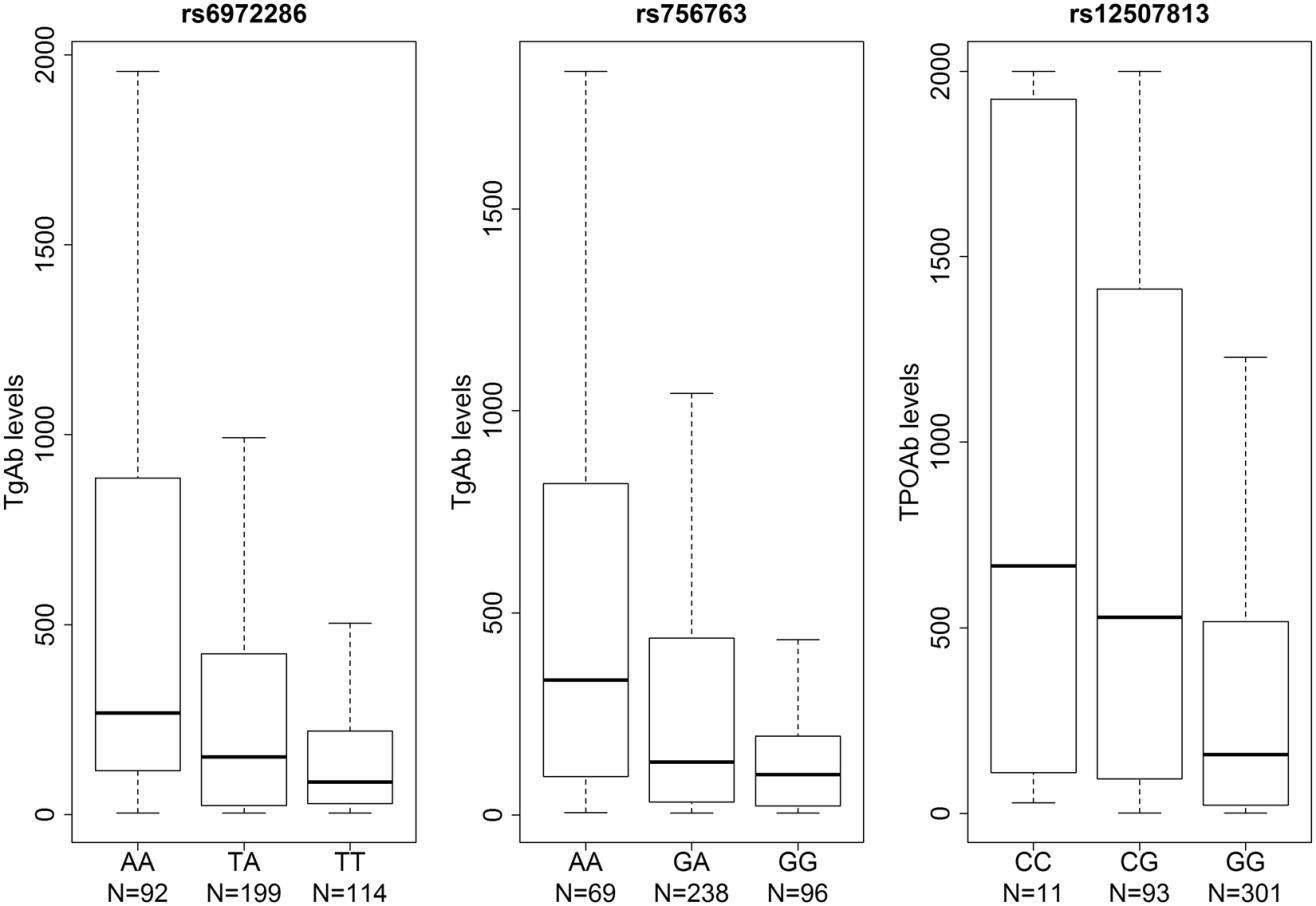
Box plots of distribution of TgAb levels per genotypes of rs6972286 and rs756763 and TPOAb levels per genotype of rs12507813.

We have detected two clear association peaks with TgAb and one with TPOAb levels at suggestive level of significance (Fig. 1a,b, respectively). Two most associated SNPs with TgAb levels were intergenic variant rs6972286 on chromosome 7, two closest genes are *ANKRD7* and *LSM8* (P = 2.34 × 10^−7^, β = 0.358, SE = 0.068 for allele A), and rs756763 inside *CA10* (P = 6.05 × 10^−7^, β = 0.406, SE = 0.08 for allele A) (Table 2). Regional association plots of these two SNPs are shown in Supplementary Fig. 1a,b, respectively. We further identified 11 SNPs associated with TgAb levels at P < 10^−5^ (Supplementary Table 1). The most associated genetic variant with TPOAb levels was rs12507813 positioned between *TRIM61* and *TRIM60* (P = 4.95 × 10^−7^, β = −0.485, SE = 0.095 for allele G) (Table 2). Regional association plot for this SNP is shown in Supplementary Fig. 2. We additionally identified 11 SNPs associated with TPOAb levels at P < 10^−5^ (Supplementary Table 1). Box plots of distribution of TgAb and TPOAb levels per genotype for all three suggestively associated SNPs are shown in Fig. 2.

**Table 2:** The most associated genetic variants with TgAb levels, TPOAb levels and both thyroid antibodies (bivariate analysis) in HT patients.

Chr	Position	SNP	Gene/nearest genes	EA	OA	EAF	β	SE	P
**TgAb levels**									
7	118325734	rs6972286	*ANKRD7* (415 kb away), *LSM8* (465 kb away)	A	T	0,469	0,358	0,068	2,34E-07
17	49749312	rs756763	*CA10*	A	G	0,467	0,406	0,080	6,05E-07
**TPOAb levels**									
4	165934574	rs12507813	*TRIM61* (36 kb away), *TRIM60* (18 kb away)	G	C	0,857	−0,485	0,095	4,95E-07
Chr	Position	SNP	Gene/nearest genes	EA	OA	EAF	P		
**TgAb and TPOAb levels (bivariate analysis)**									
5	103425505	rs13190616	*RP11-138J23.1*, *NUDT12* (527 kb away)	C	T	0,344	2,01E-06		
3	106377818	rs561030786	*DUBR* (582 kb away)	C	G	0,92	7,33E-06		
2	49250971	rs12713034	*FSHR*	A	G	0,557	7,66E-06		

Chr-chromosome, EA-effect allele, OA-other allele, EAF-effect allele frequency, β-SNP effect size, SE-standard error. P-p-value. Positions are based on the GRCh 37 build. All β (SE) values are calculated for effect allele.

### Power analysis

The estimates of study power for identification of genetic variants with a range of effect sizes and MAFs on the genome-wide level are shown in Supplementary Fig. 3. Our study had 80% power to detect common genetic variants with moderate effect sizes (for MAF = 0.45, β > 0.42; for MAF = 0.35, β > 0.43; for MAF = 0.25, β > 0.46) or with large effect sizes (for MAF = 0.15, β > 0.54; for MAF = 0.05, β > 0.73) on the genome-wide level of significance. For the top three variants from our single trait GWAS (rs6972286, MAF = 0.47, β = 0.36; rs756763, MAF = 0.47, β = 0.41; rs12507813, MAF = 0.14, β = 0.49, respectively) our study had power of 46%, 77% and 49%, respectively, to detect their association on the genome-wide level of significance.

### Bivariate GWAS analysis

The most significant SNP in the bivariate GWAS analysis was rs2056252 (P = 7.06 × 10^−7^), representing the same signal as the top TPOAb-associated SNP (rs12507813, r^2^ = 0.93), but we also identified 10 SNPs at P < 10^−5^ (Fig. 1c, Supplementary Table 1). Three out of these 10 signals were not identified in a single GWAS analyses and represent genetic variants that predispose to both antibodies. These three variants are: rs13190616 inside *RP11-138J23.1* (P = 2.01 × 10^−6^), an intergenic variant rs561030786 on chromosome 3, the closest gene is *DUBR* (P = 7.33 × 10^−6^), and rs12713034 inside *FSHR* (P = 7.66 × 10^−6^) (Table 2). Regional association plots for these three SNPs are shown in Supplementary Fig. 4a,b and c, respectively.

### Analysis of results from previously published GWAS studies

A comparison of original reports of established thyroid antibodies SNPs with results from our study showed that 5 out of 7 SNPs associated with TgAb in general population (71%), 15 out of 16 SNPs associated with TPOAb in general population (94%), and 10 out of 11 TPOAb SNPs specific for T1D patients (91%) were in the same direction (binomial sign tests: P = 0.2266, P = 0.0003, P = 0.0059, respectively) (Supplementary Table 2, Supplementary Table 3). Two SNPs were nominally associated (P < 0.05) with TPOAb levels in our study: rs879564 near *MIR7-3HG* (P = 0.0289), previously associated with TPOAb in general population, and rs4971154 inside *FCRL1* (P = 0.0236), previously associated with TPOAb in T1D patients (Supplementary Table 2, Supplementary Table 3).

In the look-up of regions spanning +/−400 kb around originally-reported SNPs we found additional associations with P < 10^−3^. As we were checking candidate-regions we chose a less stringent P-value threshold than for hypothesis free genome-wide associations. Two gene regions were associated with TgAb levels: rs368079967 between *NPSR1* and *DPY19L1* (P = 6.29 × 10^−4^) (originally reported SNP is rs323907 inside *NPSR1*) and rs11982850 near *CNPY1* (P = 8.81 × 10^−4^) (originally reported SNP is rs183893980 near *INSIG1*). Regional association plots are shown in Supplementary Fig. 5a,b, respectively. We also identified two regions associated with TPOAb levels: rs7966322 near *HNF1A* (P = 5.53 × 10^−5^) (originally reported SNP is rs662739 inside *SPPL3*), and rs4110937 near *FCRL5* (P = 4.93 × 10^−4^) (originally reported SNPs are rs11264798 inside *FCRL3* and rs4971154 inside *FCRL1*). Regional association plots are shown in Supplementary Fig. 6a,b, respectively.

## Discussion

We have performed the first GWAS analysis of both thyroid antibodies levels, TgAb and TPOAb, in a cohort of clinically diagnosed HT patients. Our study provides the first insight of genetic variants associated with thyroid autoimmunity in patients that are particularly characterized by increased thyroid antibody levels, as opposed to already performed GWAS studies in general population or T1D. We have found two suggestively associated genetic variants with TgAb levels, one with TPOAb levels and three with both thyroid antibodies. In the paragraphs below, we will briefly discuss suggestive hits with respect to thyroid autoimmunity and previously established associations. The list of all GWAS associations from these genomic regions with complex traits is shown in Supplementary Table 4.

The lead TgAb SNP (rs6972286) is an intergenic variant on chromosome 7 (Supplementary Fig. 1a). Two closest genes, *ANKRD7* (ankyrin repeat domain 7) and *LSM8* (U6 small nuclear RNA associated), are positioned approximately 415 kb and 465 kb, respectively, upstream of this variant. There are no previous genome-wide associations of this SNP or nearby genetic variants with complex traits. LSM8 is involved in splicing and maturation of RNA molecules in nucleus (www.genecards.org). *LSM8* has previously been associated with pediatric autoimmune diseases^40^. Interestingly, LSM8 (i.e. NAA38) was found to be one of the top driver oncogenes in patients with breast cancer (BC)^41^, whereas, it is already known that BC is more prevalent among patients with thyroid disorders^42^. A recent meta-analysis showed that TgAb levels are significantly increased in patients with BC^26^ while another large cross-sectional study found that BC is the most frequent extra-thyroidal malignancy in female patients with thyroid problems^24^. Although the role of TgAb antibodies in BC is not well-understood, the fact that this gene is suggested to be associated with TgAb levels in our cohort of HT patients, but also with BC, points to a possible shared underlying pathway between both traits. The other gene, *ANKRD7*, is also a good-candidate, as it has been associated with female-associated phenotypes in the UK Biobank such as ovarian cysts, ovarian problems, menopausal symptoms, but also with allergy/hypersensitivity/anaphylaxis (https://biobankengine.stanford.edu). In line with this, females usually have higher titers of thyroid antibodies^43^, while our sample consisted of 93% females.

The second most associated SNP with TgAb levels (rs756763) is intron variant inside *CA10* (carbonic anhydrase X) (Supplementary Fig. 1b). CA10 protein is a member of carbonic anhydrase family of zinc metalloenzymes and plays a role in catalysis of reversible hydration of carbon dioxide in many biological processes with specific role in brain development (www.genecards.org). GWAS and replication studies found that genetic variants inside *CA10* have been associated with asthma^44,45^, another disorder that has high co-occurrence with autoimmune thyroiditis^46,47^. Several studies investigated thyroid antibodies in patients with asthma and found significantly increased TPOAb antibodies in these patients in comparison to controls^19,20^. A high rate of hidden thyroid autoimmunity in patients with asthma may also indicate a shared genetic background between both phenotypes. With respect to that, this SNP, also associated with TgAb and TPOAb levels in bivariate analysis, may explain part of that common genetic susceptibility. Our results are in line with observation from a large comparison of GWAS studies that suggested that a cluster of immune-related diseases, including asthma and hypothyroidism, shares underlying mechanisms^48^. There are other previously established associations of *CA10*, for example, nearby genetic variant has been associated, and robustly replicated, with another exclusively female phenotype, the age at menarche, a feature that is under hypothalamic-pituitary-ovarian regulation^48–50^. Additionally, the most interesting associations of *CA10* in the UK Biobank are with thyroid problems and gynecological disorder (https://biobankengine.stanford.edu). Taken altogether, in addition to already known associations with asthma and female reproductive phenotypes, our study suggests involvement of *CA10* with thyroid autoimmunity, thus adding another layer of complexity to the role of this gene.

The top SNP from our GWAS analysis of TPOAb levels (rs12507813) is positioned between *TRIM61* (tripartite motif containing 61), 36 kb away, and *TRIM60* (tripartite motif containing 60), 18 kb away (Supplementary Fig. 2). These two genes contain RING finger domain that is usually found in proteins that interact with other proteins or DNA molecule (www.genecards.org). A proxy of this SNP (rs3733418) has been suggestively associated with obesity-related trait, monocyte chemotactic protein-1^51^, while another nearby genetic variant has been associated with insulin-related traits^52^ (Supplementary Table 4). As mentioned, thyroid antibodies are found in higher frequencies in patients with T1D^14^ while TPOAb levels are associated with homeostasis model assessment of insulin resistance (HOMA-IR)^53^ suggesting a plausible explanation of the involvement of this genomic region with both, thyroid autoimmunity and insulin-related traits. Additionally, another genetic variant (rs4691139) from this genomic region was associated with *BRCA1*-specific ovarian cancer risk^54^ pointing out again a possible genetic relationship between thyroid autoimmunity and female-specific malignancies. Finally, variants within this genomic region were also associated with neuroticism^55^ and age at menopause (*TRIM60*) in the UK Biobank (https://biobankengine.stanford.edu).

In TgAb-TPOAb bivariate GWAS analysis we identified three new signals with P < 10^−5^ that did not come-out in a single trait analyses indicating that these variants predispose to both traits: rs13190616 inside *RP11-138J23.1*, intergenic variant rs561030786 and rs12713034 inside *FSHR*.

The first variant (rs13190616) (Supplementary Fig. 4a) is a candidate cis-regulatory element (ccCRES) associated with *NUDT12* (527 kb away) and, according to ENCODE RAMPAGE expression profile, is highly expressed in thyroid tissue. Genetic variants in the 500 kb window around this SNP and *NUDT12* were already associated with depressive symptoms and depression^56,57^, thus liking yet another closely related personality trait with thyroid autoimmunity. There is a plausible explanation for this observation as TPOAb antibodies were also found to be associated with depressive symptoms and suggested to be vulnerability markers for depression^22^. Genetic variant (rs500141) near rs13190616 was also found to be suggestively associated with infection to measles^58^, proposing a role of this genomic region in immunity.

The second suggestively associated variant with both antibodies (rs561030786) is positioned 582 kb away from a long non-coding RNA gene *DUBR* (DPPA2 upstream binding RNA) (Supplementary Fig. 4b). There are no previously reported genome-wide associations with genetic variants from this genomic region. Of suggestive associations, the most interesting is association with itch intensity from mosquito bite and unattractiveness to mosquito implying to immune-related locus^59^.

The third genetic variant suggestively associated with both thyroid antibodies (rs12713034) resides inside *FSHR* (follicle stimulating hormone receptor) (Supplementary Fig. 4c). This is particularly interesting result, as a genetic variant near *FSHR* (rs1405966) has already been suggestively associated with TgAb levels in females from Croatian general population^31^, thus providing solid evidence of the enrollment of this gene with thyroid antibody development. FSHR is vital for healthy reproductive function in males and females. Several studies found genome-wide associations of variants inside or near *FSHR* with PCOS^60,61^, yet another disease with assumed autoimmune etiology that is found to be more common in patients with HT^16^ and characterized by higher frequencies of thyroid antibodies^16,17,62^. A recent review article on thyroid autoimmunity and PCOS suggested common genetic and autoimmunity-related causal factors in both diseases^16^. Moreover, *FSHR* and the above-mentioned *ANKR7* belong to gene ontology categories that are associated with sex-differentiation and reproductive traits. *FSHR* has additionally been associated with breast cancer, polycystic ovaries but also thyroid cancer (rs34201686, in moderate linkage disequilibrium (LD) with our top SNP, rs12713034, r^2^ = 0.363) and asthma in the UK Biobank. Increased FSHR expression was found in thyroid adenoma in a sex hormone-dependent manner^63^ and in thyroid cancers^64^. A body of evidence suggests the role of *FSHR* genetic variants with thyroid autoimmunity and repetitively suggests a link with many other phenotypes that are characterized by increased thyroid antibodies levels.

Additional aim of our study was to analyze if genetic variants that are associated with thyroid antibodies levels in general population and T1D patients also control the levels of thyroid antibodies in HT patients. We showed that a significant proportion of genetic variants associated with TgAb in general population, as well as genetic variants associated with TPOAb in general population and T1D patients, maintain the same direction of effects in HT patients. On the other hand, only two genetic variants, near *MIR7-3HG* and inside *FCRL1*, were nominally associated with TPOAb levels in our study, whereas, none of the selected variants was associated with TgAb levels. This implies that the genetic variants contributing to TgAb and TPOAb in general population or T1D do not necessarily have the same significance and effect sizes in HT patients. We were additionally interested to see if wider genomic regions surrounding originally reported SNPs harbor additional genetic variants that are associated with thyroid antibodies (as seen for already discussed *FSHR* genetic variants). We chose a less stringent P-value threshold of P < 10^−3^ due to hypothesis that these SNPs are true candidates for association with thyroid antibody levels. We found additional genomic regions around *NPSR1, INSIG1*, *SPPL3* and *FCLR* genes to be potentially involved in regulation of thyroid antibodies levels in HT patients (described in detail in Supplementary text). Of note, we found associations at different genetic variants that are not in LD with originally reported variants, implying that in pathological states like HT, some other genetic variants may have more important role in the regulation of thyroid antibodies than in individuals without thyroid disorders.

The main limits of our study are the modest sample size and the lack of replication cohort. Power analysis showed that our study was able to detect common genetic variants with moderate to large effects, but was underpowered to detect variants with small effects (Supplementary Fig. 3). Although Manhattan plots show clear association peaks in TgAb and TPOAb analyses, our results are suggestive. Our study had 46%, 77% and 49% power to detect association of the top three variants from single trait analyses (rs6972286, rs756763, rs12507813) on the genome-wide level of significance (P < 5 × 10^−8^). For detection of these variants on suggestive level of significance (P < 10^−6^), our study had power of 68%, 90% and 70%, respectively. To detect genome-wide significance with 80% power of our top SNP rs6972286, with the risk allele frequency of 0.47 and estimated effect size of 0.36, our sample should comprise at least 594 individuals. A replication and fine mapping of genomic regions identified in our study are required before confirming association of these genetic variants with thyroid antibodies. We were not able to perform *in silico* replication, as there are currently no HT cohorts of Caucasian origin with available GWAS data and measures for both thyroid antibodies (TPOAb and TgAb). Our primary aim was to identify HT-specific genetic variants associated with thyroid antibodies, as HT patients are distinct entity of individuals with antibody values skewed to the right of population values. This means that cohort from general population would not be suitable for replication of our results, as already discussed in the paragraph above. The next logical step in investigation of genetic background of thyroid antibodies would be to meta-analyze cohorts of HT patients and general population to discover overlapping variants predisposing to higher antibody levels in individuals in general, regardless of their thyroid function status. The main advantage of our study is the choice of samples, as HT patients are enriched for increased TgAb and TPOAb levels, thus representing individuals falling within the right end of thyroid antibodies distribution. This specific feature brings additional strength to our study. Our cohort provides an excellent resource for future meta-analyses of these traits.

In conclusion, our study aimed to identify genetic variants that are associated with thyroid antibodies levels in HT patients, which may be considered as population extremes for the thyroid antibody distribution. Our results indicate possible genetic links between thyroid autoimmunity and female disorders such as breast cancer, PCOS and phenotypes related to female-reproductive system that are all under hypothalamic-pituitary regulation. A complex interplay between female reproductive system and thyroid autoimmunity is further reinforced by involvement of two suggestively associated loci (*FSHR* and *ANKR7*) in the same gene ontology categories (sex-differentiation and reproductive traits). This study also suggests an overlapping genetic susceptibility between thyroid autoimmunity and T1D, asthma and personality traits (neuroticism and depression) which are all characterized by higher levels of thyroid antibodies in comparison to healthy individuals. We also found evidence of association of four previously reported genomic regions, albeit at different genetic variants, suggesting that thyroid antibodies levels are regulated differently in specific pathological conditions such as HT, than in individuals with normal thyroid function. The pleiotropic effects of identified genomic regions and their involvement with thyroid antibodies levels need to be replicated in a larger sample. Our study brings new insight in genetics of thyroid antibodies in HT patients and sets a firm basis for further research.

## Supplementary information

Supplementary Material

## Data Availability

The datasets of the current study are available from the corresponding author on reasonable request.
