# Supplementary material for: Genome-wide association analysis suggests novel loci underlying thyroid antibodies in Hashimoto’s thyroiditis
Source: Sci Rep. 2019 Mar 29;9:5360. doi: 10.1038/s41598-019-41850-6 (PMC6440971; doi:10.1038/s41598-019-41850-6)
Supplement: Supplementary file 1 — Supplementary Material [file 41598_2019_41850_MOESM1_ESM.pdf]

Supplementary Material

Supplement to: “Genome-wide association analysis suggests novel loci underlying thyroid antibodies in Hashimoto’s thyroiditis” (Scientific Reports).

Authors: Luka Brčić[1], Ana Barić[2], Sanda Gračan[2], Vesela Torlak[2], Marko Brekalo[2], Veselin Škrabić[3], Tatijana Zemunik[1], Maja Barbalić[1], Ante Punda[2], Vesna Boraska Perica[1]

[1]Department of Medical Biology, University of Split, School of Medicine, Split, Croatia
[2]Department of Nuclear Medicine, University Hospital Split, Split, Croatia
[3]Department of Pediatrics, University Hospital Split, Split, Croatia

Contents

Supplementary Text 2

Four candidate genomic regions associated with TgAb/TPOAb levels 2

References 3

Supplementary Tables 5

Supplementary Table 1 5

Supplementary Table 2 6

Supplementary Table 3 7

Supplementary Table 4 8

Supplementary Figures 9

Supplementary Figure 1 9

Supplementary Figure 2 10

Supplementary Figure 3 11

Supplementary Figure 4 12

Supplementary Figure 5 14

Supplementary Figure 6 15

Supplementary Text

Four candidate genomic regions associated with TgAb/TPOAb levels

Of previously associated TgAb genomic regions we found association signal near *NPSR1* (Neuropeptide S Receptor 1) that encodes G protein-coupled receptor involved in immune response[1]. Our most associated SNP resides between *NPSR1* and *DPY19L1* (Dpy-19 Like C-Mannosyltransferase 1) (Supplementary Figure 4a), of which *NPSR1* was previously suggestively associated with IgG glycosylation[2], asthma[3] and rheumathoid arthritis[1]. Our data support the role of *NPSR1* as a promising candidate in TgAb regulation.

We have also replicated association of the region around *INSIG1* (Insulin Induced Gene 1), which regulates metabolism of cholesterol, glucose and lipogenesis (www.genecards.org), with TgAb levels. However, our most associated SNP from this region resides near *CNPY1* (Canopy FGF Signaling Regulator 1) (Supplementary Figure 4b). Neither of these genes has previous established associations with relevant phenotypes.

Of previously associated TPOAb genomic regions we found signal around *SPPL3* (Signal Peptide Peptidase Like 3) that was originally discovered in T1D patients[4]. The most associated SNP from this region in our study is rs7966322 near *HNF1A* (HNF1 Homeobox A) (Supplementary Figure 5a). Interestingly, in addition to suggestive association of *SPPL3* with TPOAb levels, *SPPL3* was already associated with allergic disease (asthma, hay fever or eczema)[5], age at menopause[6] and SLE[7], all the traits that were already brought in connection with thyroid antibodies and discussed in our paper. Additionally, *HNF1A* acts as a transcription factor for several liver-specific genes (www.genecards.org) and has a substantial record of associations in GWAS catalogue. Among few most relevant to our phenotype are associations with inflammation marker alpha-1 antitrypsin levels[8], C-reactive protein[9], which was also found to be correlated with both thyroid antibodies[10], N-glycan levels[11] and epithelial ovarian cancer[12]. Taken together, our data suggest that genomic region encompassing *HNF1A* and *SPPL3* is potentially involved in TPOAb regulation in HT patients.

Finally, we also found evidence of association of the region around *FCRL3* (Fc Receptor Like 3) originally reported in T1D patients[4]. In our study, the most associated SNP (rs4110937) resides near *FCRL5* (Fc Receptor Like 5) (Supplementary Figure 5b). This region contains a cluster of *FCRL* genes belonging to the same immunoglobulin receptor superfamily with suggested role in immune system (www.genecards.org). The role of this gene in autoimmunity is further reinforced by established association with Graves' disease (GD)[13-15], another form of AITD characterized by the presence of elevated thyroid antibodies[16], and with Graves ophthalmopathy[17] that has been correlated with the presence of TPOAbs in children with GD[18]. In the case of GD where both genes, *FCRL3* and *FCRL5*, were found to be

associated with disease, additional analysis suggested that *FCRL5* was secondary to the effect of *FCRL3*[19]. Additionally, *FCRL3* has been associated with several other autoimmune diseases[20] and traits such as insulinoma-associated antigen 2 autoantibody levels in T1D[4], the presence of antibodies in SLE patients[21], rheumatoid arthritis[22] and selective IgA deficiency[23]. *FCRL3* was also found to be overexpressed in children with autoimmune thyroiditis when compared to adults[24]. Our data, together with previous reports, suggest that genomic region encompassing *FCRL* genes is a good candidate locus for regulation of TPOAb levels in patients with HT. Fine-mapping of this region in HT patients is crucial to resolve the causal variants and associated gene.

References

1 Robledo, G. *et al.* NPSR1 gene is associated with reduced risk of rheumatoid arthritis. *The Journal of rheumatology* **39**, 1166-1170, doi:10.3899/jrheum.111205 (2012).

2 Lauc, G. *et al.* Loci associated with N-glycosylation of human immunoglobulin G show pleiotropy with autoimmune diseases and haematological cancers. *PLoS genetics* **9**, e1003225, doi:10.1371/journal.pgen.1003225 (2013).

3 Demenais, F. *et al.* Multiancestry association study identifies new asthma risk loci that colocalize with immune-cell enhancer marks. *Nature genetics* **50**, 42-53, doi:10.1038/s41588-017-0014-7 (2018).

4 Plagnol, V. *et al.* Genome-wide association analysis of autoantibody positivity in type 1 diabetes cases. *PLoS genetics* **7**, e1002216, doi:10.1371/journal.pgen.1002216 (2011).

5 Ferreira, M. A. *et al.* Shared genetic origin of asthma, hay fever and eczema elucidates allergic disease biology. *Nature genetics* **49**, 1752-1757, doi:10.1038/ng.3985 (2017).

6 Day, F. R. *et al.* Large-scale genomic analyses link reproductive aging to hypothalamic signaling, breast cancer susceptibility and BRCA1-mediated DNA repair. *Nature genetics* **47**, 1294-1303, doi:10.1038/ng.3412 (2015).

7 Gateva, V. *et al.* A large-scale replication study identifies TNIP1, PRDM1, JAZF1, UHRF1BP1 and IL10 as risk loci for systemic lupus erythematosus. *Nature genetics* **41**, 1228-1233, doi:10.1038/ng.468 (2009).

8 Setoh, K. *et al.* Three missense variants of metabolic syndrome-related genes are associated with alpha-1 antitrypsin levels. *Nature communications* **6**, 7754, doi:10.1038/ncomms8754 (2015).

9 Dehghan, A. *et al.* Meta-analysis of genome-wide association studies in >80 000 subjects identifies multiple loci for C-reactive protein levels. *Circulation* **123**, 731-738, doi:10.1161/CIRCULATIONAHA.110.948570 (2011).

10 Czarnywojtek, A. *et al.* The role of serum C-reactive protein measured by high-sensitive method in thyroid disease. *Archivum immunologiae et therapiae experimentalis* **62**, 501-509, doi:10.1007/s00005-014-0282-1 (2014).

11 Huffman, J. E. *et al.* Polymorphisms in B3GAT1, SLC9A9 and MGAT5 are associated with variation within the human plasma N-glycome of 3533 European adults. *Human molecular genetics* **20**, 5000-5011, doi:10.1093/hmg/ddr414 (2011).
12 Phelan, C. M. *et al.* Identification of 12 new susceptibility loci for different histotypes of epithelial ovarian cancer. *Nature genetics* **49**, 680-691, doi:10.1038/ng.3826 (2017).
13 Khong, J. J. *et al.* Pooled genome wide association detects association upstream of FCRL3 with Graves' disease. *BMC genomics* **17**, 939, doi:10.1186/s12864-016-3276-z (2016).
14 Chu, X. *et al.* A genome-wide association study identifies two new risk loci for Graves' disease. *Nature genetics* **43**, 897-901, doi:10.1038/ng.898 (2011).
15 Zhao, S. X. *et al.* A refined study of FCRL genes from a genome-wide association study for Graves' disease. *PloS one* **8**, e57758, doi:10.1371/journal.pone.0057758 (2013).
16 Zaletel, K. Determinants of thyroid autoantibody production in Hashimoto's thyroiditis. *Expert review of clinical immunology* **3**, 217-223, doi:10.1586/1744666X.3.2.217 (2007).
17 Wu, S., Cai, T., Chen, F., He, X. & Cui, Z. Genetic associations of FCRL3 polymorphisms with the susceptibility of Graves ophthalmopathy in a Chinese population. *Int J Clin Exp Med* **8**, 16948-16954 (2015).
18 Lee, J. H., Park, S. H., Koh, D. G. & Suh, B. K. Thyroid peroxidase antibody positivity and triiodothyronine levels are associated with pediatric Graves' ophthalmopathy. *World journal of pediatrics : WJP* **10**, 155-159, doi:10.1007/s12519-014-0476-y (2014).
19 Simmonds, M. J. *et al.* Association of Fc receptor-like 5 (FCRL5) with Graves' disease is secondary to the effect of FCRL3. *Clinical endocrinology* **73**, 654-660, doi:10.1111/j.1365-2265.2010.03843.x (2010).
20 Yang, Y., Su, X., Zhang, K. & Zhou, R. The Fc receptor-like 3 gene polymorphisms and susceptibility to autoimmune diseases: an updated meta-analysis. *Autoimmunity* **46**, 547-558, doi:10.3109/08916934.2013.835804 (2013).
21 Piotrowski, P. *et al.* The FCRL3 -169T>C polymorphism might be associated with some autoantibody presence in patients with SLE in a Polish population. *Modern rheumatology*, doi:10.1007/s10165-013-0875-2 (2013).
22 Okada, Y. *et al.* Genetics of rheumatoid arthritis contributes to biology and drug discovery. *Nature* **506**, 376-381, doi:10.1038/nature12873 (2014).
23 Bronson, P. G. *et al.* Common variants at PVT1, ATG13-AMBRA1, AHI1 and CLEC16A are associated with selective IgA deficiency. *Nature genetics* **48**, 1425-1429, doi:10.1038/ng.3675 (2016).
24 Wojciechowska-Durczynska, K., Krawczyk-Rusiecka, K., Zygmunt, A., Stawerska, R. & Lewinski, A. In children with autoimmune thyroiditis CTLA4 and FCRL3 genes--but not PTPN22--are overexpressed when compared to adults. *Neuro endocrinology letters* **37**, 65-69 (2016).

Supplementary Tables

Supplementary Table 1. Results for TgAb, TPOAb and bivariate GWAS analysis in HT patients for genetic variants with $P<10^{-5}$.

TgAb levels

Chr	Position	SNP	Gene/ nearest genes	EA	OA	EAF	β	SE	P
7	118325734	rs6972286	*ANKRD7* (415 kb away), *LSM8* (465 kb away)	A	T	0,469	0,358	0,068	2,34E-07
17	49749312	rs756763	*CA10*	A	G	0,467	0,406	0,080	6,05E-07
17	13359435	rs977706	near *HS3ST3A1*	A	T	0,86	-0,513	0,105	1,42E-06
12	129621691	12:129621691:T:C	*TMEM132D*	T	C	0,472	-0,395	0,081	1,49E-06
12	152581	rs183751285	near *IQSEC3*	A	G	0,723	0,537	0,115	4,05E-06
1	213134407	rs71634696	*VASH2*	G	T	0,905	0,633	0,137	5,02E-06
5	494964	rs60857630	*SLC9A3*	C	G	0,803	0,420	0,092	5,99E-06
20	37250754	rs6026868	*ARHGAP40*	T	A	0,833	-0,464	0,103	8,56E-06
2	56335537	rs7588040	near *CCDC85A*	T	C	0,239	0,369	0,082	9,11E-06
14	40893414	rs9707549	/	C	T	0,54	-0,310	0,069	9,72E-06
8	11711473	rs1293297	*CTSB*	G	C	0,901	0,526	0,118	9,99E-06

TPOAb levels

Chr	Position	SNP	Gene/ nearest genes	EA	OA	EAF	β	SE	P
4	165934574	rs12507813	*TRIM61* (36 kb away),*TRIM60* (18 kb away)	G	C	0,857	-0,485	0,095	4,95E-07
10	51593354	rs7085433	*NCOA4, TIMM23B, TIMM23*	G	A	0,894	-0,539	0,109	1,20E-06
9	77313196	rs12551356	near *TRPM6, RORB*	G	T	0,904	-0,726	0,148	1,37E-06
1	197837567	rs72744965	near *C1orf53*	T	C	0,696	-0,380	0,080	2,78E-06
12	76219594	rs17115753	near *PHLDA1*	A	G	0,949	-0,796	0,170	4,02E-06
9	98907331	rs4077231	near *LOC158434*	A	T	0,609	-0,310	0,068	5,98E-06
3	163258976	rs6804755	near *LINC01192*	T	G	0,534	0,322	0,070	6,22E-06
3	84646348	rs113091167	near *LINC00971*	A	C	0,869	0,527	0,116	6,95E-06
9	117421666	rs79660918	near *C9orf91,*	C	T	0,919	-0,576	0,127	8,04E-06
7	46631580	rs62452723	intergenic	G	A	0,949	-0,721	0,159	8,18E-06
5	175084333	rs9313723	*HRH2*	A	C	0,9	-0,575	0,128	9,44E-06

TgAb and TPOAb levels (bivariate analysis)

Chr	Position	SNP	Gene/ nearest genes	EA	OA	EAF	P
4	165947490	rs2056252	near *TRIM61, TRIM60*	G	A	0,861	7,06E-07
7	118325734	rs6972286	*ANKRD7* (415 kb away), *LSM8* (465 kb away)	A	T	0,469	1,61E-06
5	103425505	rs13190616	*RP11-138J23.1, NUDT12* (527 kb away)	C	T	0,344	2,01E-06
17	13359435	rs977706	near *HS3ST3A1*	A	T	0,86	3,97E-06
17	49749312	rs756763	*CA10*	A	G	0,467	4,10E-06
9	77313196	rs12551356	near *TRPM6, RORB*	G	T	0,904	4,96E-06
12	129621691	12:129621691:T:C	*TMEM132D*	T	C	0,472	6,47E-06
3	106377818	rs561030786	*DUBR* (582 kb away)	C	G	0,92	7,33E-06
10	51593354	rs7085433	*NCOA4, TIMM23B, TIMM23*	G	A	0,894	7,46E-06
2	49250971	rs12713034	*FSHR*	A	G	0,557	7,66E-06

Chr-chromosome, EA-effect allele, OA-other allele, EAF-effect allele frequency, β-SNP effect size.
SE-standard error, P-p-value. Positions are based on the GRCh 37 build. All β (SE) values are calculated for effect allele.
Top associated SNPs from TgAb and TPOAb GWAS analysis, as well as SNPs from bivariate analysis that were not identified in a single GWAS analyses are highlighted.

Supplementary Table 2. Comparison of loci known to be associated with thyroid autoantibodies in general population with results from the present study.

Chr	Position	SNP	PMID of original study	Trait	Population	Original P	Nearest gene/region	EA	OA	EAF	β	SE	P	Direction of effect
6	170582064	rs4710782	29678681	TgAb	Croatian	6,16E-08	*DLL1*	C	G	0,278	-0,016	0,088	0,8554	opposite
1	65544151	rs10889518	29678681	TgAb	Croatian	1,27E-07	*JAK1*	A	T	0,057	0,068	0,160	0,6708	same
7	155106435	rs183893980	29678681	TgAb	Croatian	8,87E-07	*INSIG1*	A	G	0,122	0,068	0,118	0,5616	same
10	134943325	rs73399159	29678681	TgAb	Croatian	2,37E-07	*ADGRA1*	G	A	0,868	-0,146	0,110	0,1836	same
7	34736202	rs323907	29678681	TgAb	Croatian	3,45E-07	*NPSR1*	C	T	0,885	0,032	0,114	0,7812	same
6	158611258	rs9365994	29678681	TgAb	Croatian	6,91E-07	*GTF2H5*	C	A	0,829	0,033	0,092	0,7230	same
5	162212404	rs1288213	29678681	TgAb	Croatian	7,21E-07	*CCNG1*	A	G	0,244	0,131	0,081	0,1040	opposite
1	114173410	rs1230666	24586183	TPOAb	European	1,80E-08	*MAGI3*	A	G	0,132	0,056	0,108	0,6058	same
2	1407815	rs11675434	24586183	TPOAb	European	1,50E-16	*TPO*	C	T	0,542	-0,095	0,070	0,1778	same
6	31429927	rs3094228	24586183	TPOAb	European	3,80E-07	*HLA* region	T	C	0,762	-0,109	0,082	0,1850	same
6	32787036	rs1894407	24586183	TPOAb	European	1,20E-07	*TAP2*	C	A	0,662	0,089	0,073	0,2273	same
6	33055605	rs9277555	24586183	TPOAb	European	5,80E-07	*HLA-DPB1*	G	A	0,78	0,017	0,084	0,8400	same
6	90880393	rs10944479	24586183	TPOAb	European	4,00E-08	*BACH2*	G	A	0,802	-0,090	0,104	0,3851	same
12	112007756	rs653178	24586183	TPOAb	European	9,90E-10	*ATXN2*	C	T	0,582	0,116	0,068	0,0913	same
16	79700447	rs4889009	24586183	TPOAb	European	3,30E-07	intergenic	G	C	0,414	0,098	0,073	0,1785	same
19	4768917	rs879564	24586183	TPOAb	European	1,10E-07	*MIR7-3HG*	A	G	0,645	0,159	0,073	0,0289	same
20	51009757	rs4811340	24586183	TPOAb	European	5,60E-07	intergenic	G	C	0,689	-0,005	0,075	0,9489	opposite
2	1417244	rs2071403	24722205	TPOAb	Korean	1,30E-10	*TPO*	A	G	0,488	-0,097	0,073	0,1889	same
6	33082308	rs733208	24722205	TPOAb	Korean	4,20E-07	*HLA-DPB2*	G	A	0,829	-0,028	0,095	0,7704	same
9	104742291	rs1935377	29678681	TPOAb	Croatian	8,58E-08	*GRIN3A*	T	C	0,381	-0,011	0,078	0,8858	same
1	34438957	rs10753297	29678681	TPOAb	Croatian	5,59E-07	*CSMD2*	A	T	0,643	0,004	0,070	0,9561	same
17	31277765	rs2428361	29678681	TPOAb	Croatian	9,16E-07	*SPACA3*	C	T	0,739	0,145	0,080	0,0696	same
10	6169140	rs11256909	29678681	TPOAb	Croatian	9,61E-07	*PFKFB3*	A	C	0,698	0,076	0,074	0,3080	same

Chr-chromosome, PMID-Pubmed identification number, Original P-p-value from original study, EA-effect allele, OA-other allele. EAF-effect allele frequency, β-SNP effect size, SE-standard error, P-p-value.

Positions are based on the GRCh 37 build. All β (SE) values are calculated for effect allele.

Regions containing SNPs associated with TgAb levels at $P<10^{-3}$ in present study are highlighted.

Supplementary Table 3. Comparison of loci known to be associated with TPOAbs in T1D patients with results from the present study.

Chr	Position	SNP	PMID of original study	Trait	Population	Original P	Nearest gene/region	EA	OA	EAF	β	SE	P	Direction of effect
1	114377568	rs2476601	21829393	TPOAb	British	2,10E-05	*PTPN22*	A	G	0,114	0,124	0,112	0,2689	same
1	157661848	rs11264798	21829393	TPOAb	British	0,00035	*FCRL3*	C	G	0,525	-0,125	0,068	0,0680	same
1	157771880	rs4971154	21829393	TPOAb	British	0,0033	*FCRL1*	C	T	0,48	0,157	0,069	0,0236	same
2	191964633	rs7574865	21829393	TPOAb	British	0,01	*STAT4*	T	G	0,29	0,047	0,076	0,5380	same
2	204738919	rs3087243	21829393	TPOAb	British	0,0011	*CTLA4*	G	A	0,575	0,046	0,071	0,5145	same
4	123377980	rs2069762	21829393	TPOAb	British	0,0045	*IL2-IL21*	A	C	0,685	0,111	0,075	0,1410	opposite
6	90958231	rs11755527	21829393	TPOAb	British	9,70E-07	*BACH2*	C	G	0,529	-0,048	0,068	0,4796	same
12	111884608	rs3184504	21829393	TPOAb	British	0,003	*SH2B3*	T	C	0,574	0,118	0,068	0,0809	same
12	121229893	rs662739	21829393	TPOAb	British	0,011	*SPPL3*	T	C	0,341	0,010	0,074	0,8886	same
15	38907041	rs7171171	21829393	TPOAb	British	0,0004	*RASGRP1*	A	G	0,781	-0,001	0,087	0,9912	opposite
21	43841328	rs3788013	21829393	TPOAb	British	0,00099	*UBASH3A*	C	A	0,589	-0,089	0,074	0,2341	same

Chr-chromosome, PMID-Pubmed identification number, Original P-p-value from original study, EA-effect allele, OA-other allele.
EAF-effect allele frequency, β-SNP effect size, SE-standard error, P-p-value.
Positions are based on the GRCh 37 build. All β (SE) values are calculated for effect allele.
Regions containing SNPs associated with TPOAb levels at $P<10^{-3}$ in present study are highlighted.

Supplementary Table 4. Associations of genetic variants with other phenotypes, in the 500 kb window around lead SNP, listed in the NHGRI-EBI GWAS catalog and/or PhenoScanner.

Associated SNP	LD (R^2) with lead SNP	Position	P	Functional effect	Gene/nearest gene	Associated trait	Source	PubMed ID
**Genomic region around TgAb associated SNP rs6972286 (chr7: 118185680-119185680)**								
No genome-wide associations in this region.								
**Genomic region around TgAb associated SNP rs756763 (Chr17:51171952-52171952)**								
**Associated SNP**	**LD (R^2) with lead SNP**	**Position**	**P**	**Functional effect**	**Gene/nearest gene**	**Associated trait**	**Source**	**PubMed ID**
**Genome-wide associations:**								
rs9635759	0.0339	51.536.424	2×10^{-24}; 7×10^{-13}; 1×10^{-10}	intergenic_variant	between *RPL7P48* and *CA10*	Menarche (age at onset)	NHGRI-EBI GWAS	25231870; 21102462; 27182965
rs1392935	0.0004	51.861.769	1×10^{-8}	intron_variant	*CA10*	Bacterial meningitis	NHGRI-EBI GWAS	28928442
**Suggestive associations:**								
rs4794295	0.638	49.749.312	1×10^{-6}	intron_variant	*CA10*	Differential exon level expression of CA10 probe 3762759 in brain cortex	PhenoScanner	19222302
**Genomic region around TPOAb associated SNP rs12507813 (Chr4:164513422-165513422)**								
**Associated SNP**	**LD (R^2) with lead SNP**	**Position**	**P**	**Functional effect**	**Gene**	**Associated trait**	**Source**	**PubMed ID**
**Genome-wide associations:**								
rs17046216	0.013	165.334.552	2×10^{-8}	intron_variant	*MSMO1*	Insulin-related traits	NHGRI-EBI GWAS	22791750
rs4691139	0.1614	164.987.569	3×10^{-8}	intergenic_variant	between *TRIM61* and *LOC391713*	Ovarian cancer in BRCA1 mutation carriers	NHGRI-EBI GWAS	23544013
rs62353264	0.0018	16.516.465	4×10^{-8}	intron_variant	*TMEM192*	Neuroticism	NHGRI-EBI GWAS	27067015
**Suggestive associations:**								
rs3733418	0.8842	164.957.183	9×10^{-6}	intron_variant	*TRIM61*	Obesity related traits	PhenoScanner	23251661
**Genomic region around bivariate TgAb-TPOAb associated SNP rs13190616 (Chr5:103589804-104589804)**								
**Associated SNP**	**LD (R^2) with lead SNP**	**Position**	**P**	**Functional effect**	**Gene**	**Associated trait**	**Source**	**PubMed ID**
**Genome-wide associations:**								
rs13177365	0.0036	104.578.842	7×10^{-12}	intron_variant	*RP11-6N13.1*	Depressive symptoms (multi-trait analysis)	NHGRI-EBI GWAS	29292387
rs1363103	0.0132	104.582.136	2×10^{-9}	intron_variant	*RP11-6N13.1*	Subjective well-being	NHGRI-EBI GWAS	29292387
rs12658032	0.0058	104.568.525	6×10^{-9}	intron_variant	*RP11-6N13.1*	Depressive symptoms	NHGRI-EBI GWAS	29292387
**Suggestive associations:**								
rs500141	0.0028	103.687.094	9×10^{-7}	intergenic_variant	closest gene is *NUDT12*	Measles	NHGRI-EBI GWAS	28928442
**Genomic region around bivariate TgAb-TPOAb associated SNP rs561030786 (Chr3:106158971-107158971)**								
**Associated SNP**	**LD (R^2) with lead SNP**	**Position**	**P**	**Functional effect**	**Gene**	**Associated trait**	**Source**	**PubMed ID**
No genome-wide associations in this region.								
**Suggestive associations:**								
rs9821348	0.0011	106.570.103	5×10^{-6}	intron_variant	*LINC00882*	Perceived unattractiveness to mosquitoes	NHGRI-EBI GWAS	28199695
**Genomic region around bivariate TgAb-TPOAb associated SNP rs12713034 (Chr2:48523832-49523832)**								
**Associated SNP**	**LD (R^2) with lead SNP**	**Position**	**P**	**Functional effect**	**Gene**	**Associated trait**	**Source**	**PubMed ID**
**Genome-wide associations:**								
rs13405728	0.0113	48.751.020	8×10^{-21}	intron_variant	*STON1-GTF2A1L, LHCGR*	Polycystic ovary syndrome	NHGRI-EBI GWAS	21151128
rs2268361	0.0606	48.974.473	1×10^{-12}	intron_variant	*FSHR*	Polycystic ovary syndrome	NHGRI-EBI GWAS	22885925
rs13405728	0.0113	48.751.020	4×10^{-9}	intron_variant	*STON1-GTF2A1L, LHCGR*	Polycystic ovary syndrome	NHGRI-EBI GWAS	22885925
rs4596023	0.0111	48.728.544	2 x10-8	intron_variant	*STON1-GTF2A1L, LHCGR*	Body mass indeks	NHGRI-EBI GWAS	28892062
rs4482537	0.0069	48.825.116	3×10^{-8}	intergenic_variant	between *STON1-GTF2A1L, LHCGR* and *FSHR*	Laterality in neovascular age-related macular degeneration	NHGRI-EBI GWAS	28775256
rs2268363	0.0032	48.974.189	5×10^{-8}	intron_variant	*FSHR*	Erectile dysfunction and prostate cancer treatment	NHGRI-EBI GWAS	20932654

Positions are based on the GRCh 38 build. Linkage disequilibrium (LD) estimates are based on the 1000G phase 1 EUR reference panel (obtained using PLINK 1.9). The data was taken on 25 April 2018 from NHGRI-EBI GWAS catalog (https://www.ebi.ac.uk/gwas/)

A

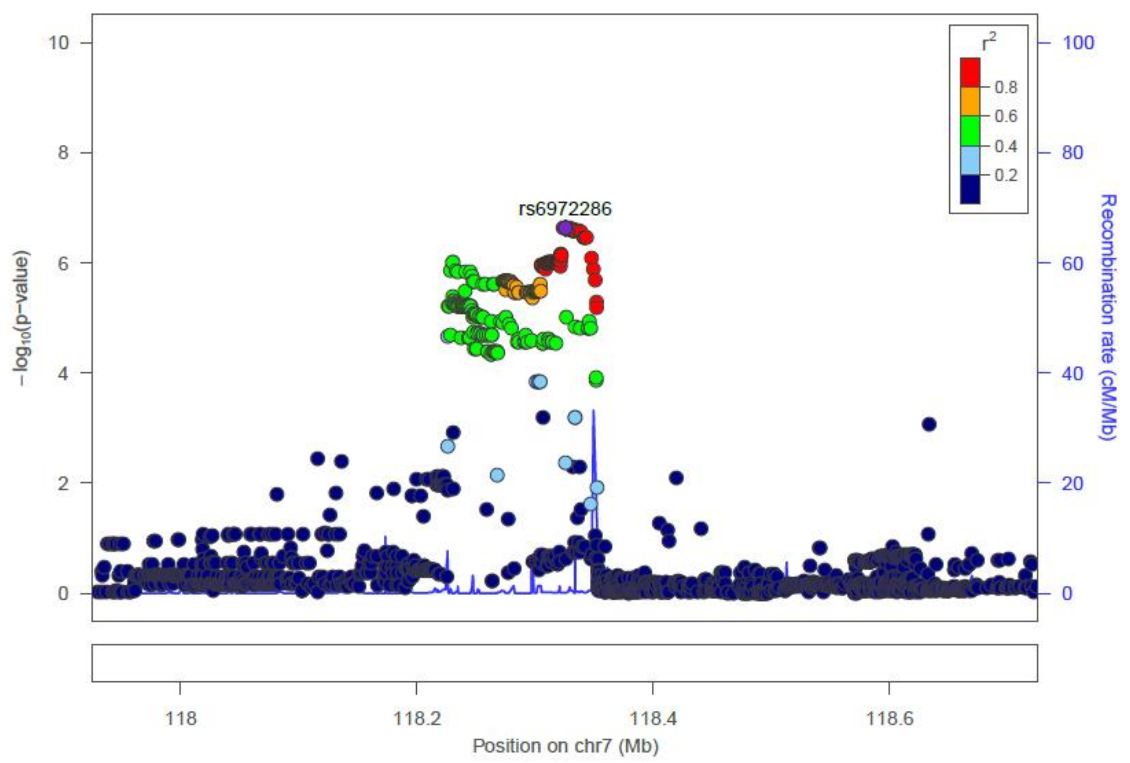

B

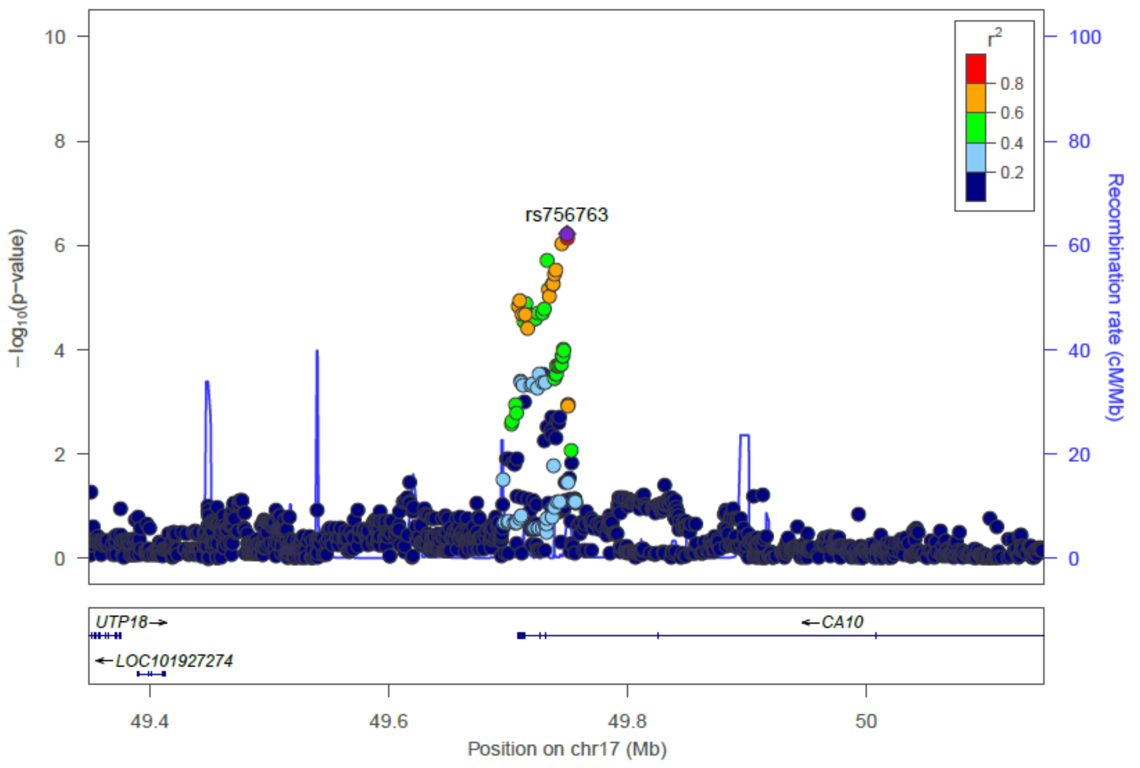

Supplementary Figure1. Regional association plots for TgAb-associated SNPs: rs6972286 (A) and rs756763 (B).

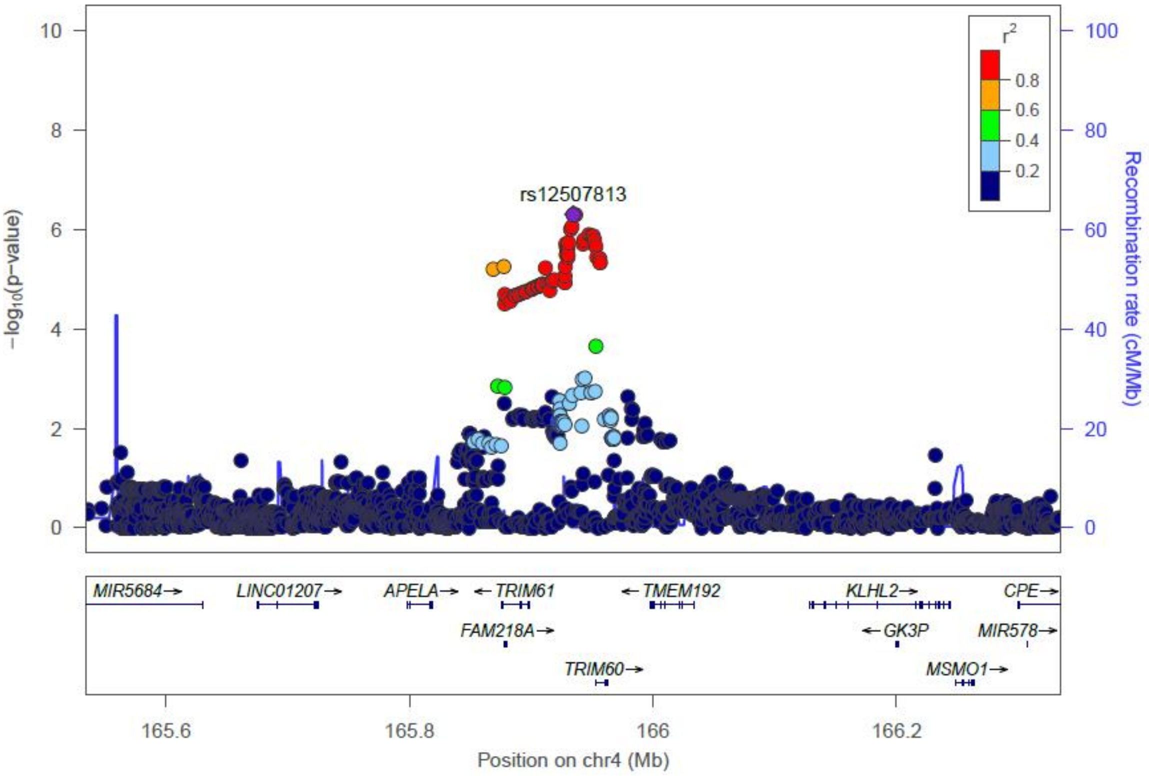

Supplementary Figure 2. Regional association plot for TPOAb-associated SNP rs12507813.

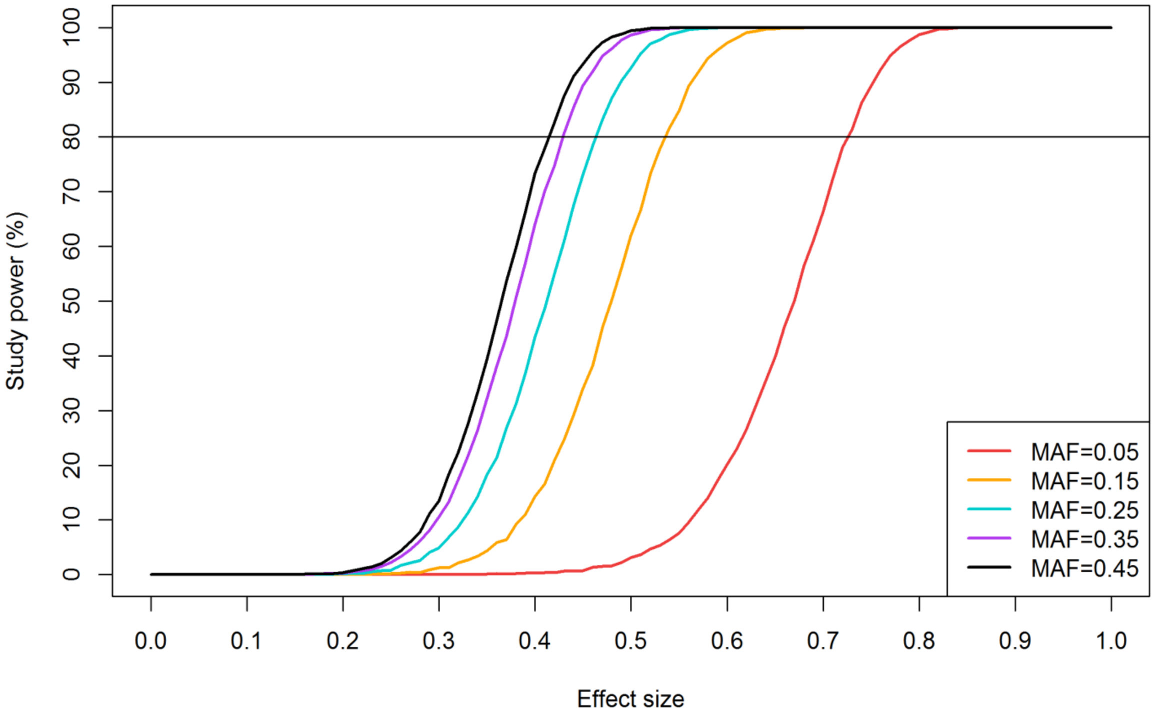

Supplementary Figure 3. Study power estimates for detection of variants with different effect sizes and minor allele frequencies (MAFs) on the genome-wide level.

A

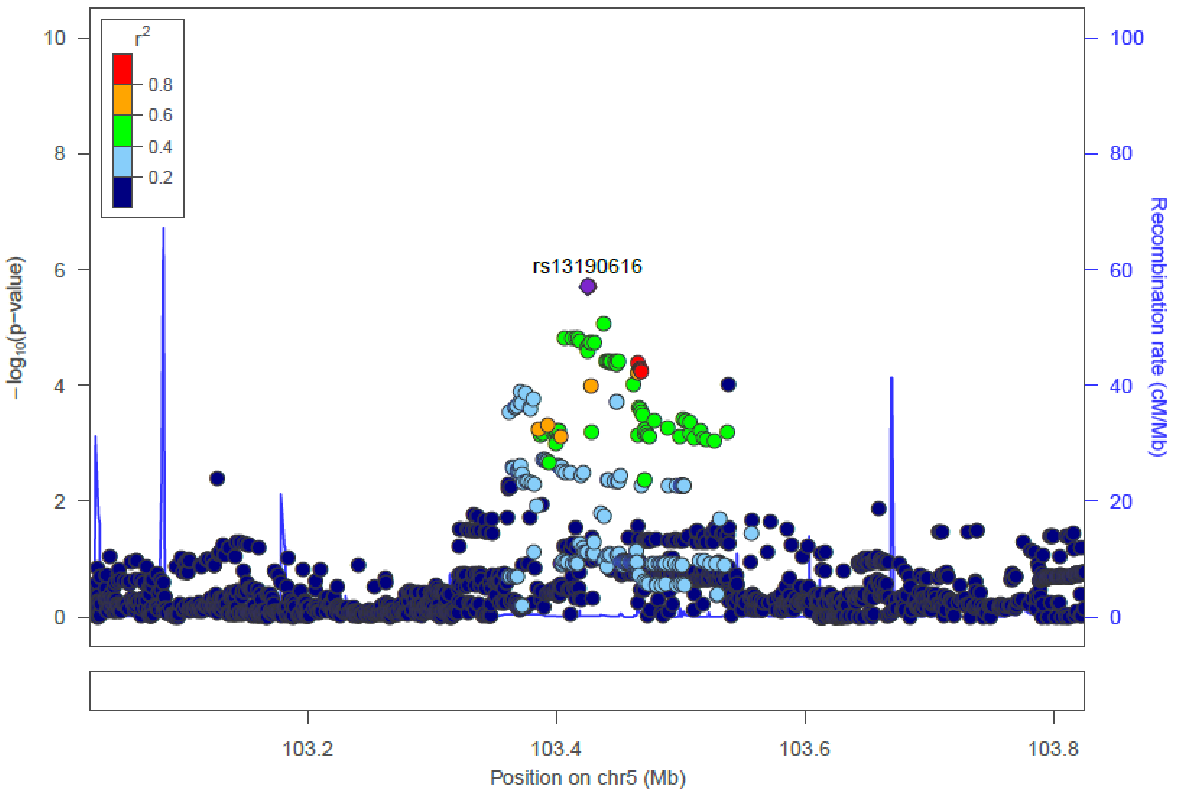
r2
0.8
0.6
0.4
0.2
rs13190616
−log10(p−value)
10
8
6
4
2
0
100
80
60
40
20
0
Recombination rate (cM/Mb)
103.2
103.4
103.6
103.8
Position on chr5 (Mb)

B

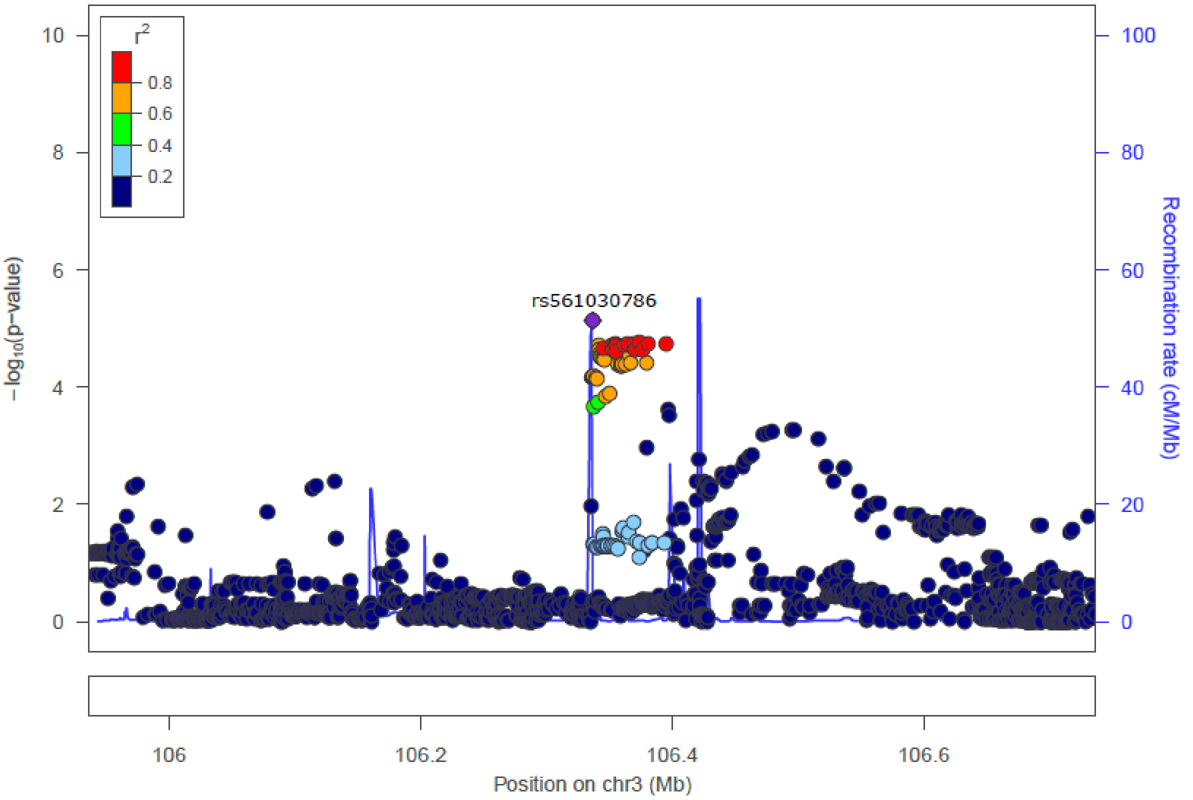
r2
0.8
0.6
0.4
0.2
rs561030786
−log10(p−value)
10
8
6
4
2
0
100
80
60
40
20
0
Recombination rate (cM/Mb)
106
106.2
106.4
106.6
Position on chr3 (Mb)

C

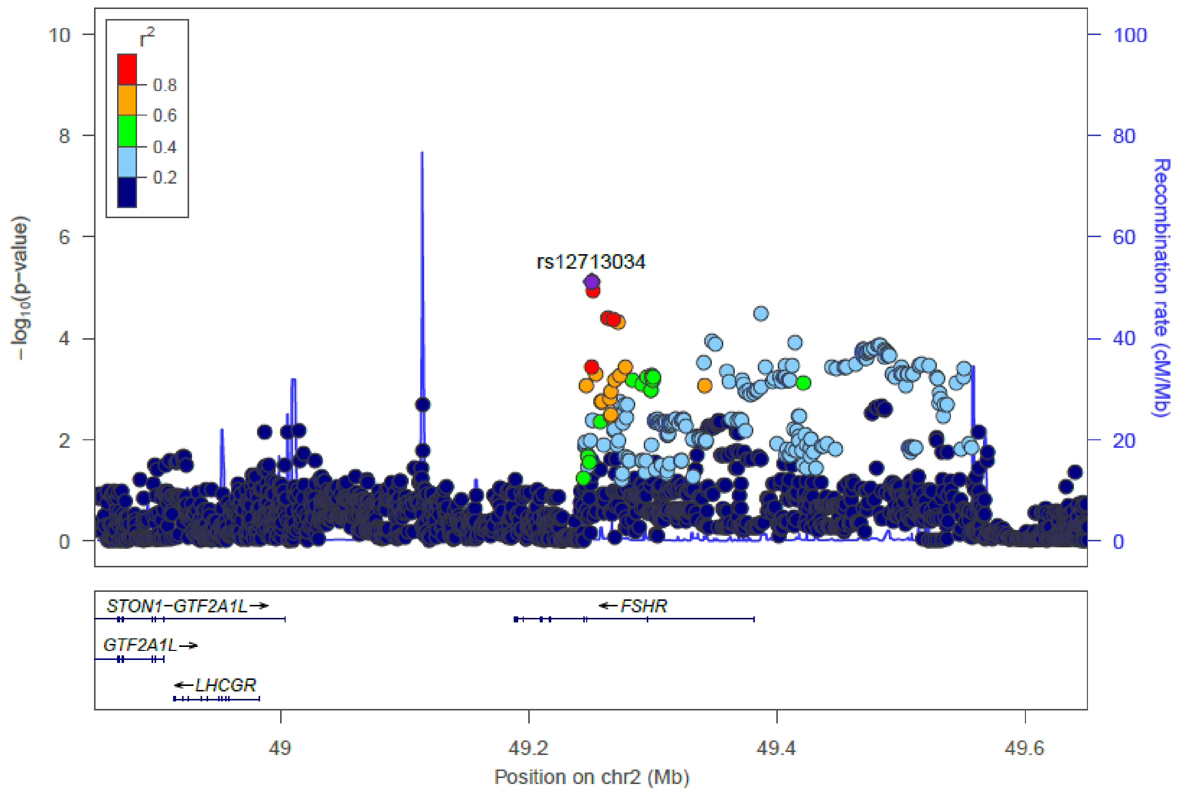

Supplementary Figure 4. Regional association plots for novel SNPs identified in bivariate GWAS analysis: rs13190616 (A), rs561030786 (B) and rs12713034 (C).

A

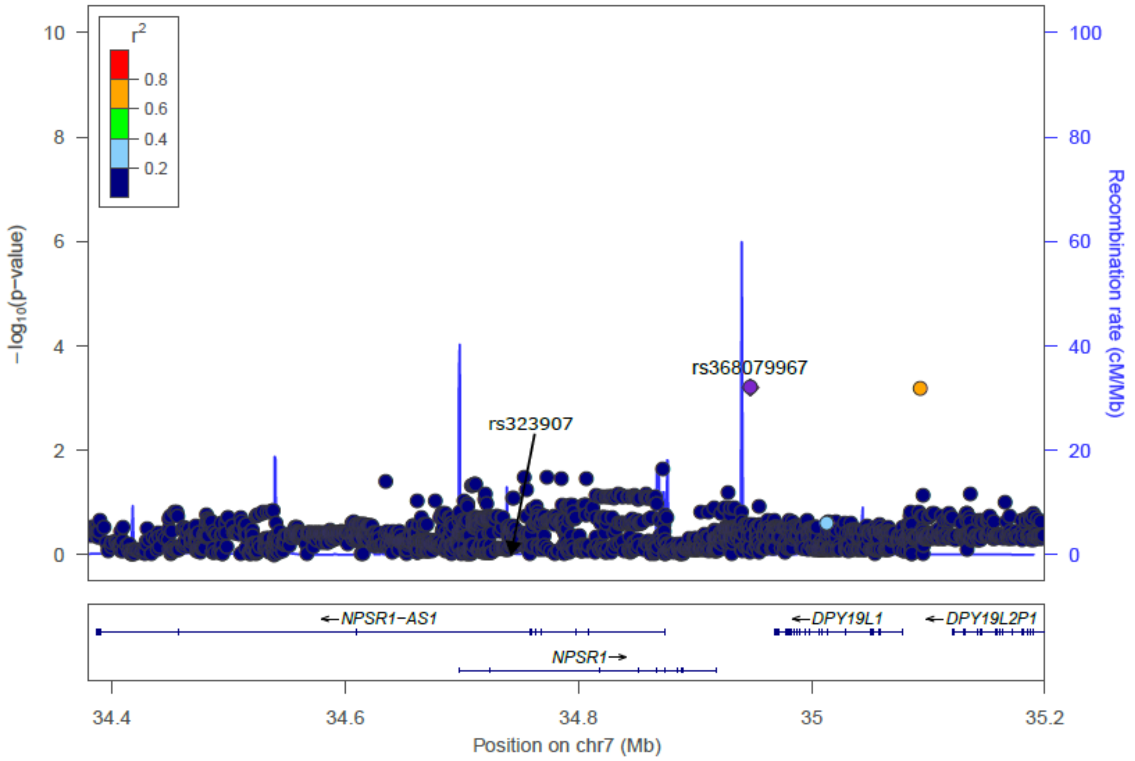

B

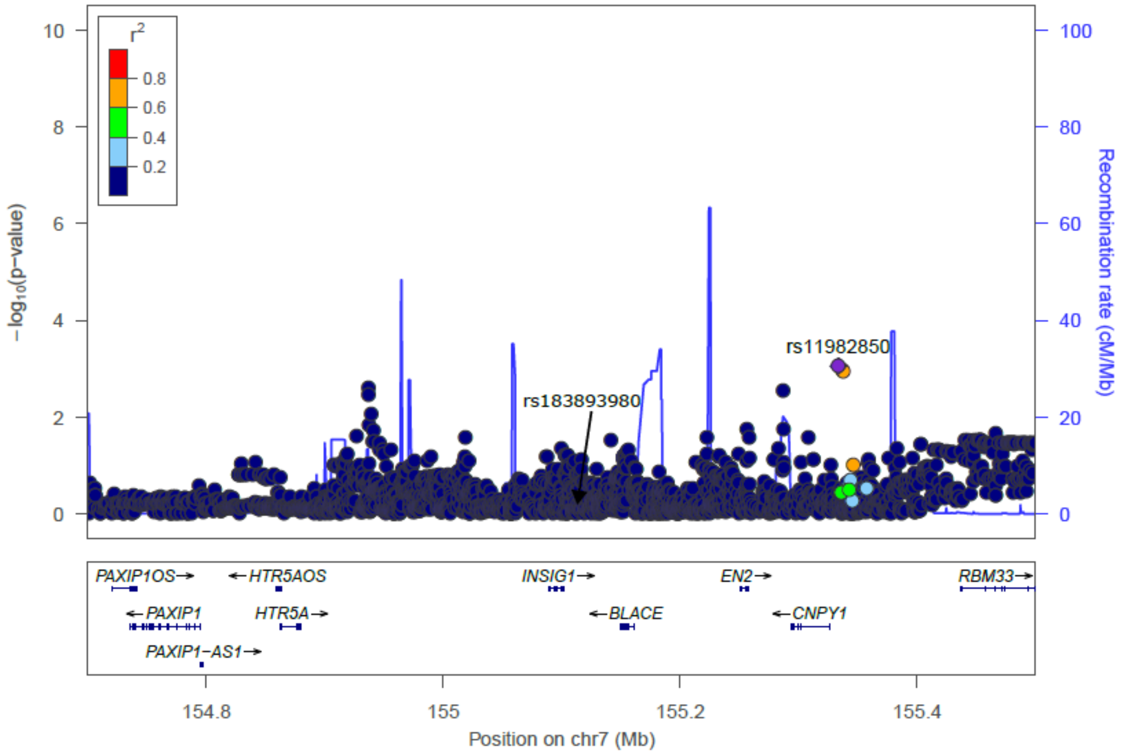

Supplementary Figure 5. The most associated SNPs in candidate gene regions with TgAb levels: rs368079967 (region surrounding originally reported rs323907 inside *NPSR1*) (A) and rs11982850 (region surrounding originally reported rs183893980 near *INSIG1*) (B) .

A

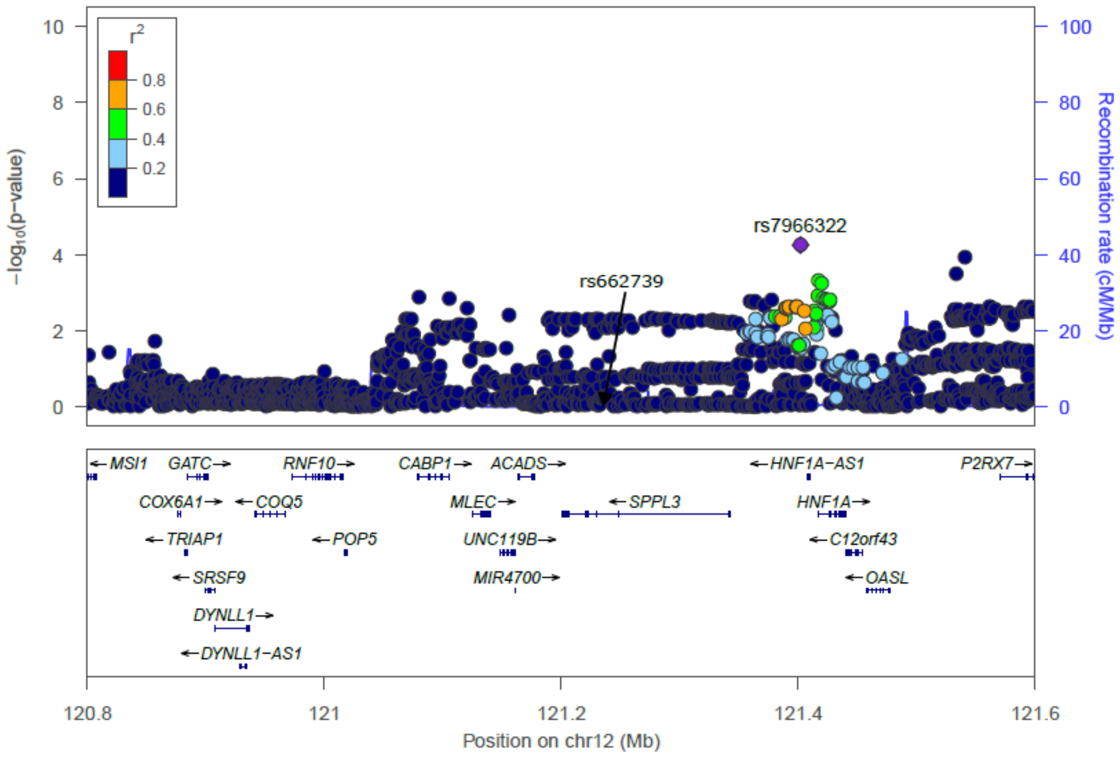

B

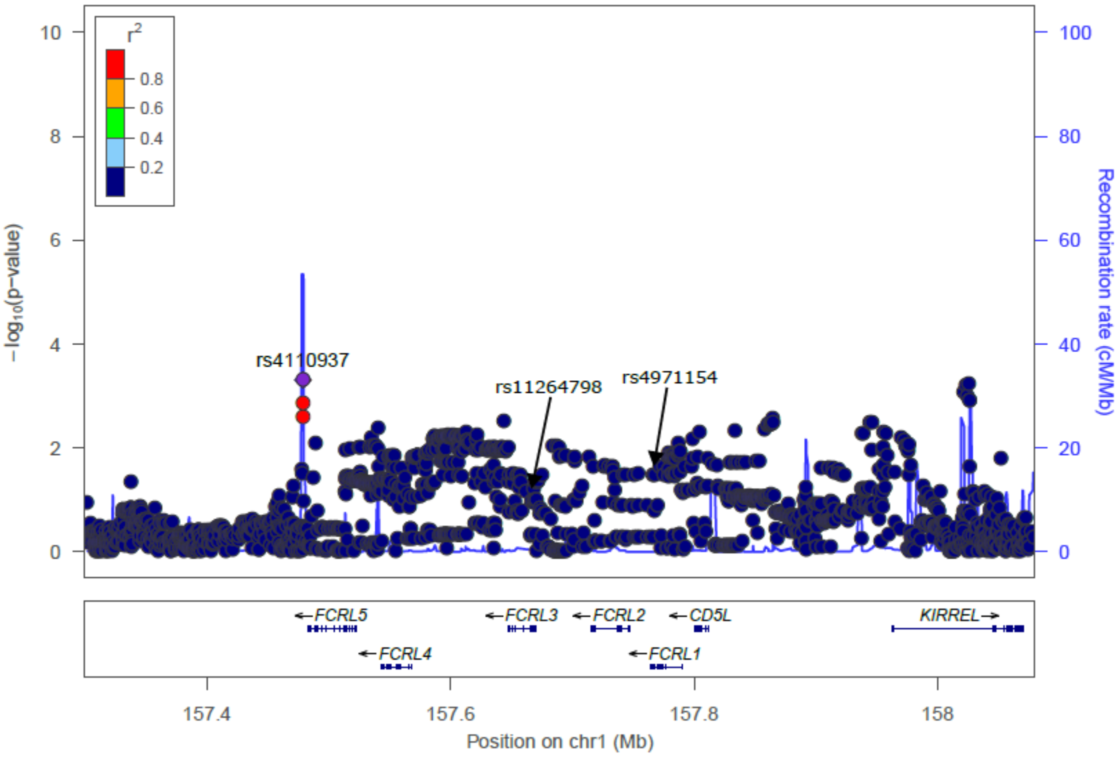

Supplementary Figure 6. The most associated SNPs in candidate gene regions with TPOAb levels: rs7966322 (region surrounding originally reported rs662739 inside *SPPL3*) (A) and rs4110937 (region surrounding originally reported rs11264798 inside *FCRL3* and rs4971154 inside *FCRL1*) (B).
